# Patient and public involvement in an evidence synthesis project: description of and reflection on involvement

**DOI:** 10.1186/s40900-024-00637-4

**Published:** 2024-10-08

**Authors:** Katie Thomson, Alex Todhunter-Brown, Marian C. Brady, Pauline Campbell, Liam Dorris, Susan M. Hunter, Donald J. Nicolson, Christine Hazelton

**Affiliations:** 1 Department of Occupational Therapy, Human Nutrition & Dietetics, Glasgow Caledonian University, Glasgow, UK; 2 Department of Nursing & Community Health, School of Health and Life Sciences, Glasgow Caledonian University, Glasgow, UK; 3 Paediatric Neurosciences, Royal Hospital for Children, NHS Greater Glasgow & Clyde, Glasgow, UK; 4 School of Allied Health Professions, Faculty of Medicine and Health Sciences, Keele University, Keele, UK; 5 Stakeholder representative, Edinburgh, UK; 6 Healthcare Improvement Scotland, Edinburgh, UK; 7 Department of Vision Sciences, School of Health and Life Sciences, Glasgow Caledonian University, Glasgow, UK

**Keywords:** PPI, Consumer, Involvement, Impact, Challenges, Evidence synthesis, Stroke, Perception

## Abstract

**Background:**

We conducted an NIHR-funded evidence synthesis project, reviewing evidence relating to interventions for perceptual disorders following stroke. This related paper describes how people with lived experience of stroke-related perceptual disorders contributed to and influenced the project, and identifies lessons for future review projects.

**Methods:**

We planned our patient and public involvement and engagement (PPIE) activities within a study protocol, described according to the domains of the ACTIVE framework; these were founded on principles for good practice in PPIE. Activities occurred across the lifespan of the project, consisting primarily of group discussions and voting to determine if there was consensus. To assess impact and individual experiences, we sought feedback using an evaluation form after each discussion, and conducted an online meeting at the end of the project to allow further reflection.

**Results:**

We recruited five people to a Lived Experience Group, including two stroke survivors and three carers. Members attended one face-to-face meeting during the development of the review. Subsequent activities were all held online due to the COVID-19 pandemic; with six online meetings, plus email interactions. Positive impacts of the Lived Experience Group on the reviews included clear definitions of key terms, selection of outcome measures, agreement on implications of review findings, and identification of research recommendations. Key challenges identified related to the complexity of the topic and challenges in the use of new online technology as a consequence of the COVID-pandemic.

**Conclusions:**

A number of lessons were learned during this project. Specific recommendations for future PPIE are to ensure that those involved have an opportunity to get to know one another, and to provide optional sessions to increase familiarity with online meeting software, clear explanations of the purpose of involvement and specific feedback after each activity. These lessons should be considered when planning the PPIE within future reviews.

**Supplementary Information:**

The online version contains supplementary material available at 10.1186/s40900-024-00637-4.

## Abstract (summary)

We reviewed research into treatments for disorders of perception after stroke. These disorders reduce a person’s ability to recognise and understand information from their senses. We involved people affected, to ensure that (i) their experiences were reflected and (ii) this complex topic was treated in a clear and understandable way. This paper discusses their involvement, the impact it had, and the lessons learnt.

We involved five people affected, within a dedicated ‘Lived Experience Group’. Two members were stroke survivors, while three were carers. Their activities spanned the whole duration of the project. An initial face-to-face meeting with the entire research team was followed by six online activities (because of COVID-19 related restrictions) and email communication.

The Lived Experience Group had a clear impact on the review. This included creating clear definitions of key terms, selecting which tests of treatment effectiveness were most important, determining the real-world meaning of the review findings, and agreeing the recommendations for stroke survivors, clinicians and researchers based on these findings. Key challenges were the complexity of the topic and terminology, the difficulty of using new technology, and the loss of personal connections arising from this mode of working.

This study demonstrates how online involvement can successfully engage stroke survivors in research. It also suggests strategies to improve future work, such as providing time and support to both practice new technologies, and for social engagement.

**Supplementary Information:**

The online version contains supplementary material available at 10.1186/s40900-024-00637-4.

## Background

PIONEER (Perceptual disorders after stroke intervention evidence review) was an NIHR-funded evidence synthesis project which explored interventions for perceptual disorders in stroke [1]. Perception is the ability to interpret and understand information from our senses (such as vision, taste, hearing, smell and somatosensation, which involves awareness of body position and temperature [2]). Perceptual disorders can affect up to 74% of persons living with stroke (referred to as stroke survivors within the remainder of this paper) [3] and cause difficulties during their everyday lives by limiting understanding of the information received through the senses [4]. The provision of rehabilitation can be challenging ; due to the range of senses involved there are a broad range of potential treatments, provided by a range of different health professionals [5]. This is particularly evident in community services where stroke survivors may receive input from more generic, interprofessional teams [6]. Stroke survivors report a lack of understanding of what perception is by healthcare professionals [7], and poor care provision [8]. The PIONEER project aimed to inform strategies to improve care by synthesising the current evidence relating to interventions for perceptual disorders in stroke survivors. It consisted of a scoping review to bring together all evidence in this field [5] and an update of an existing Cochrane systematic review [9] to synthesise evidence of effectiveness of interventions for perceptual disorders following stroke, both of which will be published in the NIHR Journals Library.

Patient and Public Involvement and Engagement (PPIE) now forms a central and integral role within healthcare research. PPIE involves the conduct of research in active partnership with patients, carers and members of the public, with contributions from members of the public influencing and shaping the research [10]. While terminology varies and can be contested, it is widely believed that when people who have lived experience of the healthcare topic of interest work together with researchers this can bring a wide range of benefits to the research [11]. For example, the research may have greater focus on what matters most to people affected by the healthcare topic, and the relevance of the research conducted may be improved. Studies concur that it may be advantageous to involve people with lived experiences of stroke in the planning and conduct of stroke research, as this may improve research relevance, impact and dissemination [12, 13] and could also have positive benefits for stroke survivors [14]. The phrase ‘stakeholder involvement’ has commonly been used to refer to the involvement of any interested person or group who is affected by a healthcare topic [15] but due to historical links to colonialism is now considered a problematic term and consensus is being sought on alternatives [16]. While we recognise that this term is not globally acceptable, consensus has not yet been reached on an alternative term and, consequently, within this paper we use ‘stakeholder’ to refer to involvement of wider groups of people (e.g. clinicians, researchers).

It is recognised that PPIE is good practice when conducting research focused on evidence synthesis, including systematic reviews [17]. PPIE in systematic reviews should adhere to wider recommendations for good practice relating to PPIE in healthcare research, including national standards [18] and guidance for reporting [19]. There is also some specific guidance relating to methods of involving people in systematic reviews such as the systematic review chapter in the Guidelines international Network [20], Cochrane’s “Involving People” resource [21] and the ACTIVE framework for describing involvement in a review [22]. However, there remain known uncertainties around best practice for, and impact of, PPIE in systematic reviews [16, 23]. Consequently, reports of examples of PPIE within specific systematic reviews can be informative, helping establish an evidence-base for PPIE in evidence synthesis. In this paper we report the PPIE within the systematic reviews conducted as part of the NIHR-funded PIONEER project. Our aim is to describe how people with lived experience of stroke-related perceptual disorders were involved in our review, to explore the impact they had on the reviews, to reflect on the process, and to identify lessons for future review projects.

## Methods

We conducted two evidence syntheses; a scoping review and a Cochrane systematic review. Methods for the conduct of these evidence syntheses are published elsewhere [5, 9]. To gain involvement of people with a range of perspectives and experiences, we worked in partnership with a Lived Experience Group, comprising a small group of volunteers who contributed throughout the project. We also worked with a group of clinicians with expertise of perceptual disorders but in this paper, we report our plans for the Lived Experience Group, and their involvement in the PIONEER evidence syntheses. We have used the GRIPP2 reporting checklist [19] to ensure the comprehensiveness of our reporting (Additional file 1) [19]. 

### Approach to involvement

Our involvement of the Lived Experience Group was founded on principles of research co-production, appreciating everyone’s contribution, making joint decisions and working together to complete the evidence syntheses [24]. We used a structured involvement approach based on the ACTIVE framework [22, 23], the Involving People resources [21], and involvement approaches used in previous systematic reviews [25]. One author on this paper (DJN) has both lived experience of perceptual problems after stroke and expertise as a health service researcher. He was a co-applicant on the NIHR-funded PIONEER project, played a key role in planning and conduct of PPIE within the project, and has contributed to all aspects of the conduct and writing-up of the project and to this paper.

We used the ACTIVE framework [22] to guide our plans to key elements, as summarised below:

#### (i) Who was involved?

We planned to form a Lived Experience Group comprising between five and eight people, aged 18-years or over, who had experience of perceptual problems following stroke, either as patients or as their carers / family members, including parents of children with perceptual problems following paediatric stroke. We sought representation of a range of perceptual disorders, but there were no formal additional criteria in place. Our decision to recruit 5–8 people was based on prior experience of the size of group that enables full involvement and inclusion of a range of views, balanced with the feasibility and resources of a project of this type.

#### (ii) How were people recruited?

Lived Experience Group members were recruited via online advertising (www.peopleinresearch.org) and through the established PPIE network of the co-applicant team. Initially, accessible information was provided on the nature of the project, and what involvement would include. Criterion checking (stroke survivor or carer with experience of perceptual issues) and further discussion with potential PPIE members took place via telephone calls which included clarification of project information (nature and duration of tasks), payment processes and communication preferences. This process also gave lived experience group members the opportunity to ask questions. We planned to continue recruitment until a minimum of five group members were confirmed. One person was thanked for their application but declined due to a conflict of interest based on their parallel involvement in a similar project.

#### (iii) What was the mode of involvement?

The ACTIVE framework describes ‘one-time’ involvement as “an approach that involves stakeholders at a specific stage of a review”, and highlights that this may occur at multiple stages in the review process; ‘continuous’ involvement is defined as “the involvement of the same stakeholders throughout the whole process” and can comprise a ‘partnership approach’ in which people contribute regularly through an advisory group [22].

We planned to combine a series of one-time activities, in which the Lived Experience Group were involved in key review-related tasks, with continuous involvement, in which group members maintained regular contact with the project team and provided oversight of the project progress. We planned to hold two face-to-face meetings, one at the start of the project and one following completion of evidence synthesis, supplemented by virtual meetings held at least once every three months. Within these meetings we planned to complete key ‘one-time’ activities, each with specific aims:

A. Defining key terms. Aim: to agree definitions and terminology and to decide how these can best be applied in the review process.
B. Prioritisation of outcome measures. Aims: to identify the ways that perceptual impairments can impact people’s lives (to inform future selection of outcome measures); and to generate a shared ranking / prioritisation of outcome domains (to select primary and secondary outcomes for the Cochrane review).
C. Interpretation of review results and identification of clinical implications. Aims: for both the scoping review and Cochrane review, to discuss the meaning of the results and reach agreement on the key findings and what these mean to people with lived experience.
D. Prioritise research recommendations. Aims: to identify research gaps and prioritise recommendations from the review findings.

For all our activities we planned to create information packs that were written to be understood by laypeople and to provide copies of presentations prior to meetings. Presentation slides were designed to be clear and uncluttered. In addition, we planned to use current low vision guidance [26] and clinical optometry experience to ensure all written materials were accessible to those with visual problems. We planned that a speech and language therapist would review all materials to ensure the accessibility of the language used.

For the project meetings we planned to use practical techniques to facilitate group member input including the use of a facilitator with expertise in lived experience involvement, agreement on ground rules based on mutual respect, and use of a timekeeper to ensure meetings ran to time, with scheduled breaks. To ensure that we provided optimal opportunities for meaningful engagement and discussion, we planned pre-meeting information packs, with the goal of enabling members to prepare in advance of meetings, to help prevent information overload during meetings, and to aid timely completion of meeting activities. For each of the activities, we planned to facilitate consensus using techniques based on the Nominal Group Technique [27] as this provides a structured method that is democratic, fosters equal participation, can help generate new ideas and ensure that everyone has an equal voice [28, 29]. We planned to invite individuals to generate, share and discuss ideas on key topics, followed by rounds of voting and ranking to explore consensus.

In addition, we planned to involve the Lived Experience Group in writing tasks, including writing of plain language summaries and planning knowledge translation activities. For these tasks we planned to communicate via email.

#### (iv) What was the level of involvement at each stage in the review process?

The ACTIVE continuum of involvement [22] describes ‘control’ as “Working in partnership with researchers, with varying degrees of control or influence over the review process. Making decisions and/or controlling one or more aspects of the review process, in collaboration with or under the guidance of the review authors.” ‘Influence’ is defined as contributions that may result in changes to the review, but without direct control. ‘Contribution’ is defined as a lower level of involvement, providing input that may indirectly affect what researchers do within the review process.

The ACTIVE framework identifies 12 stages of a systematic review. Table 1 lists the stages and summarises when and in what way the Lived Experience Group were involved in ‘one time’ activities, and the intended ‘level’ of involvement. In addition, continuous involvement was planned, with the project team maintaining contact through email and providing regular updates on project progress, and providing opportunities for the group members to *contribute* to project decisions.

### Training

To ensure that our Lived Experience Group felt equipped with the necessary skills to be able to actively participate in shared decision-making, and to provide opportunities for personal development and growth as recommended by NIHR [30], we provided basic training for the group members on systematic reviews during our first face-to-face meeting. This included an introduction to the use of evidence to answer research questions, including different types of research studies and the purpose of systematic reviews, plus additional project information such as project aims and stages and the potential role of Lived Experience Group members. If additional training needs were identified we planned to address these by signposting freely accessible online training resources (e.g. Cochrane Consumers) or by delivering teaching and mentoring sessions in response to individual needs.

### Evaluation

Using a reflective approach to working together [30], we sought feedback on the involvement and engagement of our group members using an evaluation form after each meeting (Supplementary File 1) and by asking for feedback at the end of meetings. We sought feedback on aspects that worked well and areas to improve upon, as well as what group members felt their level of influence had been within the session. In addition, at the end of the project we had a final meeting to allow group members to reflect more broadly on their experience of involvement. Meetings were audio-recorded, and researchers listened back to these to identify data (in the form of quotations) which provided reflection on the successes and challenges of involvement. The purpose of the audio-recordings was to aid us in our reflections and was not considered to be part of our research data: words and quotes reported in the reflections section of this paper are included to explicitly incorporate the reflections of the Lived Experience Group members on these topics, but no qualitative data analysis has been conducted.

### Payment

We paid group members for both their preparation time and involvement. We used UK guidance [31] to determine the level of payment provided for time spent in each activity, and paid eligible expenses. Our PPI budget amounted to 3.9% of the total project fund including to support one stroke survivor co-applicant. PPI involvement was facilitated by a senior researcher, principal investigator and research assistant.

### Ethical approval

Although PPIE activities often do not require ethical approval [32], due to the nature of our activities and our plans to collect data (e.g. in the form of votes) and record discussions (e.g. to identify important outcomes), we sought and were given approval from the GCU School of Health and Life Sciences Ethics Committee (HLS/NCH/19/021).

## Results

### Who was involved?

In addition to our co-applicant, we recruited five people with lived experience; two people were stroke survivors and three people were carers of stroke survivors. One of the carers was the parent of a paediatric stroke survivor.

### How were people involved?

Table 1 summarises the one-time activities undertaken by the Lived Experience Group members through the course of the PIONEER project with additional information on the principles of good practice used and outcomes achieved in Supplementary Files 2 and 3. The project started in January 2020, immediately prior to the start of the COVID-19 pandemic. Consequently, we only held one of the planned face-to-face meetings and all other interaction used an online meeting format (via Zoom). This led to some changes in the organisation of activities, as what had been planned as a series of practical activities, carried out face-to-face over the course of a full day, had to be changed to more frequent, but shorter, on-line meetings supplemented by off-line (email) communication. In addition to our original plans for provision of clear project materials, following feedback we developed and introduced a glossary of frequently used clinical and research terms.

*A. Definition of key terms*: A full-day meeting was held with regular breaks timetabled and discussion paced throughout the day. Initially there were introductions, presentations on the project background (including training around what a systematic review is), and discussion around the roles and responsibilities of the group members. Following this there were discussion and consensus activities around definitions and terminology; the group discussed an international definition [33, 34] of perception in vision, visuospatial, hearing, taste, smell and touch and generated a lay definition through discussion. A practical activity using slips of paper to classify and group different descriptions of perceptual disorders was conducted, and definitions of individual senses were reached through group discussion. Voting, using paper slips, was conducted after each activity to determine if there was consensus. We took care to avoid rushing through activities, ensuring everyone had an opportunity to contribute. This did mean that we did not cover everything we hoped to achieve; the prioritisation of outcome measure activities had originally been planned as part of this meeting, but we did not have time to complete this.

*B. Prioritisation of outcome measures*: During an online meeting, we formed lists of areas where members of our Lived Experience Group reported that perceptual disorders impacted their lives or those they cared for. Following the meeting the research team grouped areas of impact into similar themes and created a list of matched outcome measure categories. These were tabulated and emailed to group members who individually ranked each of the categories from 1 (most important) to 17 (least important). The rankings of the Lived Experience Group were combined with rankings of other stakeholders (clinical and research experts) and used to inform how to present data within the scoping review and the outcomes included within the Cochrane review.

*C. Interpretation of review results and identification of implications*: Four online meetings were held; the scoping review results were discussed at two meetings, the Cochrane review results at a third, and overall implications at a fourth. Prior to the first meeting, stakeholders were sent a written, lay summary which included the main results of the scoping and Cochrane review together with the presentation slides that would be used in the meeting. This information included textual summaries, bullet points of main findings together with the results of the statistical analysis. At each meeting the presentation summarised review findings before the group members were asked for feedback on three key questions: (i) What in their opinion were the key findings? (ii) What did these key findings mean to people with lived experience? and (iii) What gaps in the evidence were there? These discussions were used by the research team to incorporate different perspectives to the interpretation of review findings into publications. In particular, the group members expressed feelings of disappointment at the lack of high-quality evidence in this field, with one-member summarising this as a *“disconnect between research*,* clinical practice and the ongoing needs of stroke survivors”.*

*D. Prioritise research recommendations*: The final project tasks involved reaching consensus on the top priorities for future research relating to perceptual problems after stroke. Prior to an online meeting, group members were contacted by email and asked to submit research questions and gaps, based on their consideration of the review findings. In addition, the research team went through notes and transcripts from previous meetings and extracted any proposed research gaps, questions and recommendations. The identified research gaps were tabulated together with information on when the gap had been identified e.g. during a Lived Experience Group meeting and circulated in advance. During the meeting, previously submitted and identified research gaps were presented and group members were asked if any important questions or research gaps were missing; wording of any new questions was agreed. Nine research gaps were identified; these all related to perception as a ‘whole’, rather than to individual senses. Following the meeting, a consensus activity was carried out by email, with group members asked to rank these nine gaps on a scale from 1 to 9 with 1 being the most important area. As a prompt, group members were given the introductory statement: “A good way to think of it is to imagine you had £500,000 for a project – what would you want to spend it on?”.

**Table 1:** Summary of patient and public involvement activities conducted (supplementary file 4 provides additional information on numbers of researchers and clinical experts who also took part in these activities

Stage of review (from ACTIVE framework)	Activity	Date of activity	Mode	Number of Lived Experience Group participants	Planned ‘level’ of involvement with the Lived Experience Group (LEG)	Perceived ‘level’ of involvement
1. Develop question 2. Plan methods 3. Write & publish protocol	A. Definition of key terms	January 2020	Face-to-face meeting (full day)	2	We aimed for the LEG to have *control* over the definition of key terms and the selection of outcomes for the reviews. Through the definition of key terms (Activity A) we aimed that the LEG should *influence* the development of the search.	*Influencing*
	B. Prioritisation of outcome measures	May 2020	On-line meeting (2 h)	5		*Influencing*
		August 2020	Email; ranking exercise	4		*Controlling*
10. Interpret findings 11. Write & publish 12. Knowledge translation & impact	C. Interpret review results and identify implications	September 2020	On-line meeting (2 h)	4	We aimed for the LEG to *influence* the interpretation of findings and the text of the published reviews.	*Influencing*
		May 2021	On-line meeting (2 h)	5		*Influencing*
		November 2021	On-line meeting (2 h)	4		*Influencing*
		November 2021	On-line meeting (2 h)	2		*Influencing*
	D. Prioritise research recommendations	December 2021	On-line meeting (2 h)	2		*Contributing*
		February 2022	Email; ranking exercise	4		*Influencing*

Italics has been used to indicate that this is terminology taken from the ACTIVE Framework and the level of involvement indicated by PPIE members

### Reflections on involvement

#### Throughout the project

Evaluations collected after each meeting highlighted what those involved thought had been good, and not so good, about the activities and the manner in which they were able to contribute. During a final reflection on their engagement in this project, members of the Lived Experience Group described their involvement using the words *interesting*,* rewarding*,* relevant*,* supportive* and *educational*.

Throughout the project ‘perception’ was a challenging concept for people to understand and was described as being a field full of “*complicated jargon*”. People found it difficult to distinguish which stroke related problems directly related to their sense of perception and problems which related more generally to life after stroke. However, the lay definition agreed at the start of the project was considered useful, and was repeatedly referred to during later meetings. During review of written materials, such as the plain language summary, the group members were cognizant of trying to help others understand this complex topic. One member commented

“*I’ve tried to simplify things a bit*,* and I’ve struggled with the way that certain things have been worded….”* (Lived Experience Group member).

People involved appreciated feedback and evidence of direct impacts were perceived positively. For example, the people involved were aware that their input had impacted on the review outcome measures, with one member stating that it was good to see that their input was *“embedded in what’s been done”*, while another spoke passionately about the importance of their input, and the potential impact that their involvement may have:

“*I really would like to think that something would come out of this study*,* in terms of just getting basic things at the beginning when somebody has a stroke….*” (Lived Experience Group Member).

The use of voting to confirm consensus, which was used in different ways throughout the project, was perceived positively and helped the Lived Experience Group feel valued and listened to:

*“*[I] *felt listened to and free to share my opinions.* [It was] *an open and supportive environment*,* people interested and contributions valued with different activities*,* voting was well organised*”. *(Lived Experience Group member)*

In exploring the results of the reviews, the group members became aware that most research studies measured aspects of perceptual function as the main outcome, while in contrast our main outcomes related to ability to carry out and participate in activities. This demonstrated the direct impact of the Lived Experience Group, who felt that they had an element of control over the selection of outcomes for the Cochrane review.

#### Face-to-face meeting

The Lived Experience Group reflected positively on the one face-to-face meeting, which they felt provided a good opportunity to meet each other and the wider project team. It also provided time to discuss the role of the group and share perspectives and experiences; however, some of the terminology relating to perception was considered challenging and the original agenda was too ambitious for the time available:

***“****Use of technical language and jargon but that’s maybe unavoidable*,* a briefing on technical language would be good”.* (Lived Experience Group member)

*“Participants found the day challenging (although enjoyable). There wasn’t enough time to work through all items on the agenda and if [more] information had been sent in advance this might have helped. It was a lot to cover by including six senses … a larger voice for those with a lived experience was required”.* (Researcher).

#### Impact of COVID-19 pandemic and online meetings

The majority of this project took place during the COVID-19 pandemic, and stakeholders acknowledged that they were struggling with use of technology, the move to virtual meetings and with the impact of lockdown on their lives more generally. We provided additional support to enable Lived Experience Group members to use online communication platforms such as Zoom and spent five two-hour online meetings covering what we had originally planned to cover in one face-to-face meeting.

The lack of face-to-face interaction and move to online meetings was perceived negatively and the people involved would have preferred face-to-face meetings:

*“Had it been non-COVID times it would have been much better*,* as we’d actually have been able to meet. Well*,* Zoom*,* and the like types of meetings are good*,* they’re not the same as face-to-face interaction.”* (Lived Experience Group member).

*“…it’s been a bit of really hard battle to actually do anything and everything was online…you don’t get that personal touch*,* you know the patient impression you don’t pick them up on the body language. You know*,* for me*,* it’s difficult… But*,* face-to-face I’m happy with”.* (Clinical expert)

“*Internet connection wasn’t sufficient for a few participants*”. (Lived Experience Group member)

Furthermore, the perceptual impairments of some of the people involved caused practical problems with online interaction:

*“I did have some problems with some of the charts*,* and the way that some of the information was laid out and screen…. that was quite difficult for me. And obviously that’s because [of] my vision…”* (Lived Experience Group member).

## Discussion

### Summary of findings

Our Lived Experience Group were an integral part of the research team and were actively involved throughout this NIHR-funded evidence synthesis project focussed on perceptual disorders following stroke. They contributed to key tasks at pre-planned stages of the review using a range of different methods of involvement including face-to-face and online meetings, and in voting and ranking activities.

The involvement of the Lived Experience Group impacted on the reviews in number of direct ways. Specifically;

- consensus was reached on definitions – and lay definitions - of key terms and these were used throughout the project including in the search strategy, selection of studies, data synthesis and interpretation of findings;
- important outcomes were identified and prioritized, directly impacting the selection of outcomes of interest for the Cochrane review;
- reflections on the review results generated a list of implications and research recommendations which were integrated into the discussions in the review reports, ensuring that published implications reflected the views of people with lived experience.

Further, members of the Lived Experience Group read and commented on drafts of plain language summaries, leading to clarifications in wording and content of the final published summaries.

Reflecting on their involvement, group members revealed a number of positive feelings, both in terms of personal interest and reward and also in terms of a perceived beneficial impact on the evidence syntheses. However, members of the group did find that aspects of involvement were challenging, particularly the move to online meetings during the COVID-19 pandemic. The visual perceptual impairments of people with stroke caused difficulties during online meetings, with some people struggling with the format in which information was presented.

### Lessons learned

The people involved were asked what they would change to improve their experiences of involvement. Specific recommendations for improving involvement in future projects were:

1. **Make sure people know one another.** The group members highlighted that it can feel more intimidating to speak with people you don’t know. They emphasized the importance of taking time to get to know each other. In relation to meetings that involved professionals as well as people with lived experience, a pre-meeting of patient / carer members was recommended, providing people with an opportunity to share experiences and build understanding with other members of the group.
2. **Support on-line meetings.** While a preference for face-to-face meetings was made, the advantages of on-line meetings (e.g. no travel) were recognized. Having optional sessions to increase familiarity with the on-line format and be able to receive support on how to use various features within on-line meeting software was recommended.
3. **Provide clear explanations of the purpose of involvement.** People involved in this project understood the value of their involvement when they later saw the review. However, they recommended that it would be beneficial if this was more clearly explained, enabling them to understand why their input was required and how it was going to impact the research that was conducted.
4. **Give detailed feedback after each activity**. Despite the research team attempting to provide feedback on the impact of involvement, this was done informally and in an ad hoc manner. Reflections from the people involved highlighted that it would have been beneficial if this feedback had been provided in a more structured and specific way; for example, after each meeting providing a clear statement of (i) what was discussed/achieved/agreed during the last activity and (ii) the impact or influence that this will have on the research.

### Strengths and limitations

Our approach to PPIE used current UK guidance, ensured suitable reimbursement for time involved, used an established method to plan and describe how people were involved, and used relevant reporting guidelines [19]. We also reflected on the involvement process, by collecting written feedback after meetings and discussing what had and what had not gone well. However, we only involved five people with lived experience, arguably meaning that our findings were shaped by the particular experiences of this group. For some meetings this meant that only a small number of Lived Experience Group members were in attendance (for example only 2 attended the in-person meeting). Therefore, a breadth of views is unlikely to have been captured however all information recorded at meetings (including decisions) were circulated to the full Lived Experience Group for comment. Our planned level of involvement for our Lived Experience Group members was achieved for all activities (Table 1) with the exception of definition of key terms where members felt they had influence (captured via feedback forms) rather than control. This may have been due to the limited number of Lived Experience Group members able to attend the in-person event.

Very few feedback forms were returned by stakeholders following the meetings. The research team reflected that this lack of formalised feedback may be due to the number of project meetings that were taking place within a short period of time, and that stakeholders were prioritising preparation for and attendance at the meetings over requests for paperwork.

The COVID-19 pandemic and the move to online / remote interaction only limited the activities and impacted involvement in this project. The people with lived experience highlighted the pandemic as a key limitation in their ability to engage and contribute to activities. However, having been involved in activities both before and during lockdowns, the people involved in this project are uniquely placed to compare and contrast the different modes of engagement.

Only one person with lived experience of stroke contributed to the writing of this paper. Ideally all members of the Lived Experience Group would have been invited to participate in forming this paper. Unfortunately, the research team had insufficient time or funding resources to facilitate this. For future studies we should ensure that we plan appropriately for PPIE contributions to paper writing beyond the end-date of the project.

This paper describes and reflects on one experience of patient and public involvement in an evidence synthesis project. It is important that lessons learned from this project are considered alongside lessons from other evidence synthesis projects. The need to develop clear guidance to support best practice in the involvement of people with lived experience, and other stakeholders, in evidence syntheses has been internationally recognised. An international consortium is currently developing guidance for stakeholder involvement in healthcare syntheses [16].

The work being conducted by this international consortium includes a suite of evidence syntheses [35] which will bring together lessons learned from evidence synthesis projects around the world. Furthermore, in September 2023, Cochrane demonstrated its commitment to supporting best practice in the co-production of evidence syntheses, launching a Cochrane Co-production Methods Group [36]. These initiatives should support important advances in knowledge relating to optimal ways of involving people in future evidence syntheses.

## Conclusions

Five people with lived experience of perceptual problems after stroke contributed to a review of evidence on this topic. They were involved in one face-to-face meeting and, due to the COVID-19 pandemic, a series of remote activities, including online meetings, voting, prioritisation and reviewing written materials. Their involvement impacted on definitions of key terms used in the review, the selected outcome measures, the research recommendations and the writing and presentation of the reviews. Reflecting on their involvement, people described this as interesting, rewarding, relevant, supportive and educational, but also challenging. Key challenges related to the complexity of the area of research and the move to online meetings. Specific recommendations arising from this project and informing future PPIE are: ensure that the people involved have an opportunity to get to know one another; and provide support for online meetings, clear explanations of purpose of involvement and specific feedback after each activity.

## Electronic supplementary material

Below is the link to the electronic supplementary material.

Supplementary Material 1

Supplementary Material 2

Supplementary Material 3

Supplementary Material 4

## Data Availability

The datasets generated and/or analysed during the current study are not publicly available, but are available from the corresponding author on reasonable request.
