# Supplementary material for: Patient and public involvement in an evidence synthesis project: description of and reflection on involvement
Source: Res Involv Engagem. 2024 Oct 8;10:102. doi: 10.1186/s40900-024-00637-4 (PMC11462723; doi:10.1186/s40900-024-00637-4)
Supplement: Supplementary file 1 — Supplementary Material 1 [file 40900_2024_637_MOESM1_ESM.docx]

**PIONEER: record of stakeholder involvement**

**Lived Experience Group / Clinical Expert Group**

Date of involvement: ________________

| **I:** | tick |
| --- | --- |
| Have experience of a perception problem after stroke |  |
| Am a healthcare professional |  |
| Other (please state) |  |

I would describe my role at this point in the project as:

| **Role** | I am: | tick |
| --- | --- | --- |
| **Leading** | Initiating the review; taking lead responsibility for carrying out and completion of review. |  |
| **Controlling** | Working in partnership with researchers, with varying degrees of control or influence over the review process. Making decisions and/or controlling one or more aspects of the review process, in collaboration with or under the guidance of the review authors. |  |
| **Influencing** | Stating, commenting, advising, ranking, voting, prioritising, reaching consensus. Providing data or information which should directly influence the review process, but without direct control over decisions or aspects of the review process. |  |
| **Contributing** | Providing views, thoughts, feedback, opinions or experiences. Providing data or information which may indirectly influence the review process. |  |
| **Receiving** | Receiving information about the systematic review, or results of the review. |  |

| Thinking of your involvement in this meeting / event: | |
| --- | --- |
| Do you think your involvement changed or influenced anything?  If so, in what way? |  |
| What was good? |  |
| What was not so good? |  |
| What would you change? |  |

Do you have any other comments?

**Thank-you for completing this form.**

**Notes on development of this Evaluation Form**

This evaluation form was developed by members of the author team, based on the published ACTIVE Framework (Pollock 2019). The goal was to capture who was involved, at what stage in the review they were involved, their perceived level of involvement at that stage, and any feedback about how the involvement activities had gone and how the involvement had shaped the review.

A similar evaluation form was developed and used in another NIHR-funded systematic review project (Todhunter-Brown 2024). The stakeholders involved in that project provided feedback on earlier versions of an evaluation form, supporting the development of the form used for this project.

Pollock 2019. Pollock A, Campbell P, Struthers C, Synnot A, Nunn J, Hill S, et al. Development of the ACTIVE framework to describe stakeholder involvement in systematic reviews. J Health Serv Res Policy. 2019 Oct;24(4):245–55.

Todhunter-Brown 2024. Todhunter-Brown A, Booth L, Campbell P, Cheer B, Cowie J, Elders A, et al. Strategies used for childhood chronic functional constipation: the SUCCESS evidence synthesis. Health Technol Assess 2024;28(5). https://doi.org/10.3310/PLTR9622
