# Supplementary material for: Patient and public involvement in an evidence synthesis project: description of and reflection on involvement
Source: Res Involv Engagem. 2024 Oct 8;10:102. doi: 10.1186/s40900-024-00637-4 (PMC11462723; doi:10.1186/s40900-024-00637-4)
Supplement: Supplementary file 2 — Supplementary Material 2 [file 40900_2024_637_MOESM2_ESM.docx]

**Supplementary File 2**

Summary of key principles of good practice used throughout this project:

- Working in partnership with our lived experience group using published guidance available.
- Following a pre-determined plan for involvement, which detailed the decisions that the lived experience group members were to contribute to, and the planned level of involvement (control over decision making).
- Using principles of research co-production where everyone’s contributions were valued and decision making occurred jointly.
- A lived experience group member was a co-applicant and author of this paper.
- We considered the information and training needs of our lived experience group members to facilitate their involvement.
- We tailored activities to maximise our lived experience group members contribution.
- We used agreed ground rules in our meetings.
- Our lived experience group members received payment and expenses.
- We asked for feedback on the way lived experience group members were involved and acted on the information provided.
- Note taking during discussions was supplemented by audio-recording, which was used to support meeting notes. Meeting notes were circulated to all lived experience group members aiding transparency and providing opportunities for any further clarifications.
