# Supplementary material for: Patient and public involvement in an evidence synthesis project: description of and reflection on involvement
Source: Res Involv Engagem. 2024 Oct 8;10:102. doi: 10.1186/s40900-024-00637-4 (PMC11462723; doi:10.1186/s40900-024-00637-4)
Supplement: Supplementary file 3 — Supplementary Material 3 [file 40900_2024_637_MOESM3_ESM.docx]

**Supplementary file 3: Outcome of involvement activities**

| **Activity** | **Outcomes of involvement** |
| --- | --- |
| A. Definition of key terms | Original technical definition of perception: “specific mental functions of recognizing and interpreting sensory stimuli”.  Lay definition agreed: “Processing and understanding information from the senses”.  The Lived Experience Group helped us to create a lay definition of perception that would be used throughout the project. |
| B. Prioritisation of outcome measures | Original list of potential outcomes (from the project protocol):   - Activities of daily living - Standardised measures of perceptual function. - Ability in extended activities of daily living - Quality of life and social isolation - Depression and anxiety. - Discharge destination or residence after stroke. - Adverse events - Economic outcomes - Carer burden   Expanded and prioritised list created:   1. Activities of Daily living (ADL) 2. Extended ADL (eADL) 3. Social activities and participation 4. Psychological and mental health 5. Quality of Life (QoL) 6. Mobility navigation and safety 7. Sensation, cognition, motor ability, attention 8. Impact on rehabilitation 9. Perceptual function 10. Impact on family friends and carers 11. Paediatric specific – measure of development, education etc 12. Discharge destination 13. Feasibility, acceptability etc 14. Adverse events 15. Ability to compensate using other skills 16. Neurological function 17. Economic outcomes   Our project proposal listed a number of key outcomes of interest for the scoping and Cochrane Review. Following feedback from our Lived Experience Group including a ranking exercise these outcomes of interest were changed. |
| C. Interpret review results and identify implications | Key implication points, arising uniquely from Lived Experience Group input:   1. The need to provide more information to stroke survivors about any perceptual disorders they have. 2. To increase awareness and understanding of perceptual disorders following stroke among the public and healthcare professionals involved in stroke care. 3. The need for frequent, face-to-face support, tailored to the needs of the individual with perceptual disorders after stroke 4. The importance of opportunities for stroke survivor and carers to meet and talk with others affected, as this could help address the associated psychological and emotional impacts. |
| D. Prioritised research recommendations | To be published separately. |
