# Supplementary material for: Patient and public involvement in an evidence synthesis project: description of and reflection on involvement
Source: Res Involv Engagem. 2024 Oct 8;10:102. doi: 10.1186/s40900-024-00637-4 (PMC11462723; doi:10.1186/s40900-024-00637-4)
Supplement: Supplementary file 4 — Supplementary Material 4 [file 40900_2024_637_MOESM4_ESM.docx]

**Supplementary File 4: Involvement of Lived Experience Group alongside the Clinical Expert Group and Research Team**

Who took part (n=)

| Activity | Stage of  Review | Constituent  activities | Lived  Experience Group | Clinical  Expert Group | Research Team |
| --- | --- | --- | --- | --- | --- |
| 1. Definitions of key terminology | 2. Plan methods  4. Develop search  6. Select studies | n/a – only one meeting held | 2 | 3 | 9 |
| 2. Outcome measure prioritisation | 2. Plan methods | a) Online meeting to  identify measures | 5 | 0 | 2 |
|  |  | b) Email ranking  process | 4 | 3 | 8 |
| 3. Interpretation of scoping review results* | 9. Analyse data  10. Interpret findings | c) Meeting 1 – initial  results | 4 | 4 | 7 |
|  |  | d) Meeting 2 – final  results | 5 | 3 | 6 |
| 4. Interpretation of Cochrane review results* | 9. Analyse data  10. Interpret findings | n/a – only one meeting held | 4 | 1 | 11 |
| 5. Clinical implications* | 10. Interpret findings  11. write and publish review | n/a – only one meeting held | 2 | 2 | 8 |
| 6. Research recommendations* | 10. Interpret findings  11. write and publish review | e) Identify research  gaps | 2 | 2 | 9 |
|  |  | f) Agree research  priorities | 3 | 4 | 8 |
| Continuous involvement |  |  |  |  |  |
| Project oversight | All stages |  | 5 | 4 | 11 |

*The meeting included a section where members of the Lived Experience Group met separately with the PPI lead to discuss their thoughts and interpretation. These were then fed back to, and discussed by the whole team
